# Variation of Cholinesterase-Based Biosensor Sensitivity to Inhibition by Organophosphate Due To Ionizing Radiation

**DOI:** 10.3390/s90705580

**Published:** 2009-07-14

**Authors:** Miroslav Pohanka, Miroslav Koch

**Affiliations:** 1 Centre for Advanced Studies and Department of Toxicology, Faculty of Military Health Sciences, University of Defense, Trebesska 1575, 50002 Hradec Kralove, Czech Republic; 2 VF Company, Namesti Miru 50, 679 21 Cerna Hora, Czech Republic; E-Mail: miroslav.koch@vf.cz

**Keywords:** biosensor, cholinesterase, acetylcholinesterase, radiation, organophosphate, paraoxon, nerve agents

## Abstract

A cholinesterase based biosensor was constructed in order to assess the effects of ionizing radiation on exposed AChE. Although the primary objective of the experiment was to investigate the effect of ionizing radiation on the activity of the biosensor, no changes in cholinesterase activity were observed. Current provided by oxidation of thiocholine previously created from acetylthiocholine by enzyme catalyzed reaction was in a range 395–455 nA. No significant influence of radiation on AChE activity was found, despite the current variation. However, a surprising phenomenon was observed when a model organophosphate paraoxon was assayed. Irradiated biosensors seem to be more susceptible to the inhibitory effects of paraoxon. Control biosensors provided a 94 ± 5 nA current after exposure to 1 ppm paraoxon. The biosensors irradiated by a 5 kGy radiation dose and exposed to paraoxon provided a current of 49 ± 6 nA. Irradiation by doses ranging from 5 mGy to 100 kGy were investigated and the mentioned effect was confirmed at doses above 50 Gy. After the first promising experiments, biosensors irradiated by 5 kGy were used for calibration on paraoxon and compared with the control biosensors. Limits of detection 2.5 and 3.8 ppb were achieved for irradiated and non-irradiated biosensors respectively. The overall impact of this effect is discussed.

## 1. Introduction

Organophosphates, organophosphonates (henceforth jointly abbreviated as OP) and carbamates are typical anticholinergic compounds which are widely known as pesticides or nerve agents [1]. Their common toxicological pathway is based on multiple mechanisms, including increased oxidative stress and modification of blood proteins; however, their main mechanism of action is esterification of active serine in serine hydrolases [2]. Especially, blood acetylcholinesterase (AChE; EC 3.1.1.7), butyrylcholinesterase (BChE; EC 3.1.1.8) and (β-glucuronidase; EC 3.2.1.31) are typical markers of intoxication [3]. The most important toxicology pathway of OP as well as carbamates is based on inhibition of AChE in neurosynapses. AChE terminates neurotransmission through neurosynaptic cleft in both central and peripheral nervous system; accumulated acetylcholine may overstimulate nicotinic and/or muscarinic receptors [4]. Symptomatic manifestation of intoxication typically consists from bronchospasms, bradycardia, miosis, lacrymation, diarrhoea, salivation; moreover, typical symptoms of CNS nicotinic and muscarinic receptors overstimulation would also occur: confusion, coma, agitation and/or respiratory failure [5].

The sensitivity of cholinesterases to the inhibitory effects of OP, carbamates and some natural toxins such as aflatoxins and drugs suitable for treatment of symptomatic manifestations of Alzheimer’s disease was found to be applicable to the construction of biosensors for the assay of the given inhibitors [6–8,34]. Though the principle of the assay is the same, OPs, in contrast to Alzheimer’s disease drugs and aflatoxins, are irreversible inhibitors. Many methods are widely available for the construction of cholinesterase based biosensors (CBBs). Amperometric [9–10], potentiometric [11–13] and optical, including non-linear optics [14–16], biosensors are typical examples of CBBs. Voltammetric techniques are applicable for the assay of multiple analytes [17–19]. The common voltammetric principle of cholinesterase activity measurement is typically based on electrochemical oxidation of nascent thiocholine coming from acetylthiocholine [20,21]. Presence of OP in analyzed sample leads to the stopping of the mentioned reactions.

The sffects of ionizing radiation on cholinesterase activity *in vivo* has not been widely studied. Krokosz *et al*. proposed that that total activity of AChE in human erythrocytes would change due to X-rays [22]; moreover, Dimberg *et al*. found an increase of AChE activity, as well as nerve growth factor protein in mice brain after exposure to X-rays [23]. The mentioned studies on *in vivo* impact on the body and changes of AChE activity would be on a transcription level. The presented study is aimed at following of changes of intercepted AChE on an electrochemical strip during exposition to radiation. Durability of biosensors under radiation and the impact on analytical parameters is considered. Moreover, prediction of achieved results to a viable body will be made.

## 2. Results and Discussion

Biosensors were constructed as described in the Experimental section. Thirty five freshly prepared biosensors were separated into seven groups. Another ten strips were used in experiments without immobilization of AChE or any other modification. Biosensors were kept under standard laboratory conditions and all radiation as well as measurements were carried out under these conditions.

**Figure 1.**
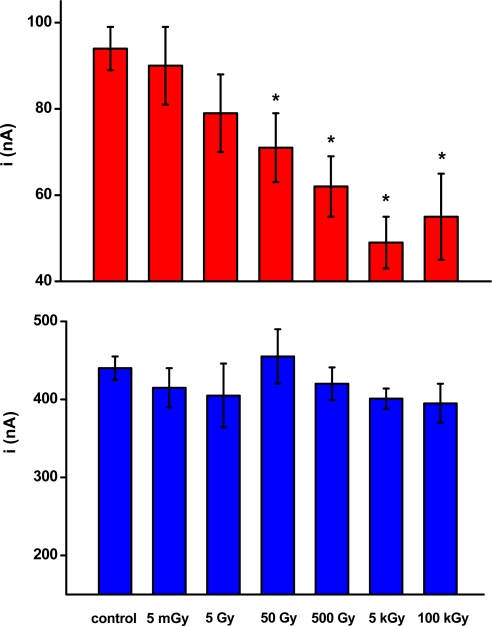
The figure depicts variation of AChE activity (as current) in biosensors due to ionizing radiation. The blue columns indicate current provided by biosensors without any inhibition. The red columns represent current provided by biosensors after exposition to paraoxon 1 ppm. Error bars indicate standard deviation (n = 5). Asterisks indicate significant difference against control at probability level P = 0.05 (ANOVA with Scheffe test).

Twelve groups (n = 5) of biosensors were irradiated with doses of 5 mGy to 100 kGy. Two groups were kept as a control. Two groups of biosensors were prepared for one radiation dose. The first group was used to investigate the activity of AChE in the absence of paraoxon, while the second group was used to investigate the AChE activity in the presence of paraoxon. Data obtained are summarized in Figure 1.

We expected a decrease of immobilized enzyme activity as the ionizing radiation exceeded the typical mortal dose. We suggest that the lethal dose of radiation is individual and strongly depends on the time of exposure. A dose of 1 Gy within one hour causes radiation sickness, vomiting, diarrhea, and hemorrhage. Incidence of cancer would be abrupt in the future. Doses of 2–5 Gy lead to acute symptoms and extensive mortality. However, no significant differences in the control biosensors were found, even when biosensors were extensive irradiated. Measured current fluctuated in a range from 395 to 455 nA. The values were overlaid within their standard deviations and no difference or correlation to radiation dose was found. The fact would be surprising when the typical effects on the body are considered [24]. The data indicate good stability of AChE when exposed to ionizing radiation and wide stability of immobilized AChE would be also expected [25]. The overall stability of biosensors was previously estimated [10].

Though the immobilized AChE displayed no specific changes in activity after exposure to ionizing radiation, a surprising result was achieved when paraoxon was assayed. A 1 ppm solution of paraoxon was assayed by biosensors previously exposed to radiation as well as the control ones. Residual activity of AChE of around 22% (current 94 ± 15 nA) remained when the activity of the control biosensors was considered. A quite different phenomenon arose when the irradiated biosensors were used for assay purposes. The residual current provided by irradiated biosensors was slightly lower when the dose of radiation was lower than 50 Gy. However, the decrease was not significant. The current provided by biosensors consequently exposed to radiation and paraoxon was 79 ± 9 nA. On the other hand, doses of 50 Gy – 100 kGy potentiated AChE to be extensively inhibited by paraoxon. The lowest current (the most extensive inhibition) was found at biosensors irradiated by 5 Gy. It was 49 ± 6 nA, representing a residual activity of 11%. In order to find out whether the residual activity would decrease more, biosensors irradiated by 100 kGy were used. The achieved current after inhibition by paraoxon was 55 ± 10 nA. It seems that radiation above 5 kGy has no further effect on the sensitivity of AChE-based biosensors towards the representative inhibitor paraoxon. We should imention that the dose of 100 kGy is quite high. There were e.g., found changes in the color of the ceramic support of the electrochemical strip and overall blackening of the plastic holder used in the experiments.

As described above, an increased sensitivity of AChE to the inhibitory effects of paraoxon was found *in vitro* using electrochemical biosensors. It should be clearly stated that the effect would be also expected to occur *in vivo* when a viable body is simultaneously exposed to ionizing radiation and an organophosphate. In a wide context, this fact could be also contributing to the commonly known cumulative health risks [26]. Confirmation of the health effect of simultaneous exposure may be expected in the future.

Since ionizing radiation was found to be able to influence the sensitivity of AChE to inhibition by the organophosphate paraoxon, calibration was considered for the subsequent experiments. As mentioned above, deterioration of the AChE-based biosensors due to ionizing radiation was not observed. To the contrary, an improvement of analytical parameters would be expected due to the increased sensitivity towards the inhibitory effect of the organophosphate paraoxon.

**Figure 2.**
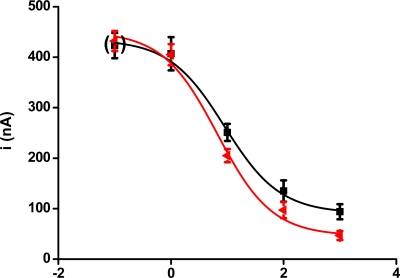
The figure depicts calibration curve provided by biosensors with AChE exposed to paraoxon–ethyl solution. The red curve was achieved by performance of biosensors previously stimulated to dose of ionizing radiation 5 kGy. The black curve represents assay of paraoxon by biosensors without any exposition to radiation. Points at brackets were achieved by assay of blank (no paraoxon). Error bars indicate standard deviation (n = 5).

Calibration curves for the biosensors are presented in Figure 2. Two calibrations were performed. The first curve presented in the figure was achieved for the performance of the biosensor without any stimulation by radiation. The second curve was obtained when paraoxon was assayed with a biosensor irradiated by a 5 kGy radiation dose. The calibration curves were found to differ; however, the difference was gradual as the concentration of paraoxon increased. The limit of detection was calculated as a point on calibration curve responding to triple de error of the control (no paraoxon) i.e., S/N = 3. The lowest limit of detection for biosensors without stimulation by radiation was 3.8 ppb. The irradiated biosensor provided a lower limit of detection when compared with the non-irradiated ones: 2.5 ppb. The effect of radiation appears to be most effective in the range of higher paraoxon concentration. On the other hand, a 49% shift of the limit of detection was found. This correlated appropriately with data presented in Figure 1.

The above mentioned calibrations point to improved calibration curves provided by biosensors challenged by ionizing radiation. It also represents a possible route in the continuous effort to improve assays where AChE is a promising recognition element [27]. It seems that pre-activation of biosensors would be an appropriate way to improve available biosensors.

The phenomenon that sensitivity of AChE to organophosphates inhibition would change after exposition to ionizing radiation is quite a surprising fact. No relevant studies are currently available in scientific databases. Both the substrate and inhibitor were applied after radiation therefore an impact of these molecules on the researched phenomena would be excluded. The active site of AChE is quite stable and modification(s) in the ester and/or anionic sites should be evidenced by a depletion of enzyme activity [28,29]. Instead, access of organophosphates into a cavity with an active cleft would be a reason for the variation of AChE sensitivity to inhibition by an organophosphate [30,31]. However, this fact should be further clarified in the future. The effect of increased inhibitory potency of paraoxon after biosensor irradiation would be only estimated in the present study. A possible explanation could be found in a study by Weik *et al*. [35]. They found extensive sensitivity to X-rays of the His-440 residue in the active site of electric eel AChE. An impact of radiation would be also expected on the surface Glu-306, catalytically active Glu-327, and disulfide Cys-254-Cys-265 [35]. Dissociation of disulfides and modification of surface structural amino acids would be responsible just for increased penetration into the active site.

## 3. Experimental Section

### 3.1. Biosensor Construction

Original electric eel AChE (2,000 U/mg) was purchased from Sigma-Aldrich. AChE was suspended in phosphate buffered saline with 0.1% glutaraldehyde and albumin 1 mg/mL up to a final activity of 5 U/μL (further suspension). An electrochemical sensor strip with platinum working electrode (1 mm diameter, dot shaped) localized in circles of Ag/AgCl reference and platinum auxiliary electrodes was purchased from BVT (Brno, Czech Republic). The strips were washed by ethanol and consequently distilled water prior to use. After drying, the working electrode was overlaid by 2 μL of suspension and allowed to dry in a fridge. The freshly prepared biosensors were used immediately or kept at 4 °C.

### 3.2. Exposition to Ionizing Radiation

The certified laboratory in the VF Company (http://www.vf.eu) was used for irradiation of the biosensors. An emitter with ^238^Pu was used as a source of radiation. Position and distance from the emitter to the irradiated biosensors was scaled by an adjustable system and observed with a digital camera system. The emitter was validated by calibration with dosimeters, Geiger counters and other radiation detectors. Biosensors were fixed horizontally and irradiated by one of the following doses: 5 mGy, 5, 50, 500 Gy, 5 kGy, or 100 kGy during one hour period, except for the 100 kGy dose which was applied overnight (12 hours) due to the low power of the emitters. Biosensors measurements were performed approximately 30 minutes after irradiation. Biosensors were kept in SATP conditions when radiated and/or while measurements were performed.

### 3.3. Electrochemical Evaluation of Enzyme Activity

**Scheme 1.**
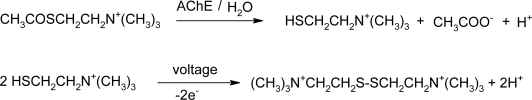
Principle of electrochemical evaluation AChE activity.

Activity of cholinesterases would by typically evaluated by a photometric method [32]. Here, an electrochemical principle based on chronoamperometry was used. The main advantage of electrochemical biosensors is the simple manipulation of solid state exposed AChE and no interference by coloring of materials due to radiation, such as would be typical for plastic cuvettes used in the photometric assay. Prior to measurement, a fresh solution of paraoxon (paraoxon-ethyl; analytical standard; Labor Dr. Ehrenstorfer-Schafers; Augsburg, Germany) in 5% aqueous isopropanol was prepared. The influence of organic solvents was optimized previously and results clearly suggested the need to avoid concentrations of isopropanol higher than 5% [10]. Solutions of paraoxon were prepared freshly every day of the experiment due to the known spontaneous hydrolysis of paraoxon in water. Measurements were started by immersing the biosensor into 1 mM acetylthiocholine chloride (ATChCl) in PBS. The concentration of ATChCl as well as the overall experiment design were optimized in a previous study [10]. An EmStat (Palmsens, Houten, The Netherlands) electrochemical analyzer was used throughout the experiments; applied voltage was adjusted up to 450 mV for all experiments as proposed in previous experiments [10,33–34]. The electrochemical signal rises quickly [9,33]; however, the actual value of the current was read after 30 seconds of current stabilization. Inhibition by paraoxon and calibrations were performed in a similar way as described above. Paraoxon was injected into the reaction cell just at the moment when the abovementioned current was measured i.e., paraoxon was in medium at the same time as the ATChCl. It was left to interact for another 30 seconds and the current was measured again. Each biosensor was used for only one measurement since the inhibition by paraoxon is irreversible. Moreover, control biosensors were also used only once due to better reproducibility of experiments. All measurements were performed five times. The principle of the electrochemical assay is depicted in Scheme 1.

### 3.4. Data Processing and Statistics

Measurements performed by the EmStat device were controlled with the PSLite (Palmsens) software. The software allowed measurement of actual current values. The obtaned data were processed in OriginPro 8 (OriginLab Corporation; Northampton, MA, USA). Significance of observed phenomena were assessed by ANOVA with a Scheffe test. Measurements were repeated five times. Limit of detection was estimated as a point on calibration scale responding to 3x of the control measurement standard deviation (S/N = 3).

## 4. Conclusions

Cholinesterase based biosensors are an intensively researched group of biosensors. The current applications being widely published by many authors suggest promising performance and expected commercialization. Experiments presented here concerned the variation of cholinesterase activity when irradiated. The primary goal of the experiments was to assess the stability of immobilized AChE. Though something like a “biodosimeter” was expected, AChE was found to be stable, even under high doses of radiation. An unexpected effect was found when the pesticide paraoxon was assayed using the irradiated biosensors. The inhibitory efficacy of paraoxon was potentiated by irradiation. Though the achieved data are only preliminary, the positive effect on the assay is readily recognized.
